# Sox2 maintains epithelial cell proliferation in the successional dental lamina

**DOI:** 10.1111/cpr.12729

**Published:** 2019-11-19

**Authors:** Eun‐Jung Kim, Seo‐Yoon Jung, Zhaoming Wu, Sushan Zhang, Han‐Sung Jung

**Affiliations:** ^1^ Division in Anatomy and Developmental Biology Department of Oral Biology Research Center for Orofacial Hard Tissue Regeneration Brain Korea 21 PLUS Project Oral Science Research Center College of Dentistry Yonsei University Seoul Korea

**Keywords:** cell proliferation, Claudin10, Laminin5, Sox2, successional dental lamina

## Abstract

**Objectives:**

The successional dental lamina is the distinctive structure on the lingual side of the vertebrate tooth germ. The aim of this study was to investigate the relationship among Sox2, Claudin10 and laminin5 and the role of Sox2 in successional dental lamina proliferation during vertebrate tooth development.

**Materials and Methods:**

To understand the successional dental lamina, two types of successional tooth formation, that in geckos (with multiple rounds of tooth generation) and that in mice (with only one round of tooth generation), were analysed.

**Results:**

Unique coexpression patterns of Sox2 and Claudin10 expression were compared in the successional dental lamina from the cap stage to the late bell stage in the mouse tooth germ and in juvenile gecko teeth to support continuous tooth replacement. Furthermore, Laminin5 expression was shown in the cap stage and decreased after the bell stage. Upon comparing the epithelial cell cycles and cell proliferation in successional dental lamina regions between mouse and gecko molars using BrdU and IdU staining and pulse‐chase methods, distinctive patterns of continuous expression were revealed. Moreover, Sox2 overexpression with a lentiviral system resulted in hyperplastic dental epithelium in mouse molars.

**Conclusions:**

Our findings indicate that the regulation of Sox2 in dental lamina proliferation is fundamental to the successional dental lamina in both species.

## 1 INTRODUCTION

Mammals such as humans and pigs have two generations of teeth (diphyodont).^1^ The dental lamina of these species starts to disintegrate when the first generation reaches the late bell stage after initiation of the second‐generation tooth bud.^2^ Reptiles such as snakes and lizards have multiple generations of tooth replacement throughout their lives (polyphyodont).^2^ The dental lamina remains intact, enabling continuous tooth replacement. Some species, such as mice and chameleons, have a rudimentary successional lamina that regresses, as has been observed on the lingual side of the first functional teeth in mice (monophyodont).^2^, ^3^, ^4^ Tooth buds are initiated from the dental lamina, a stripe of stratified epithelium.^5^ Tooth replacement in vertebrates is initiated from the end of the dental lamina, known as the successional dental lamina.^6^

The transcription factor Sox2 is essential for stem cells and progenitor cells to maintain pluripotency,^7^, ^8^ and ablation of Sox2 in mice leads to early mortality after implantation.^9^ Sox2 is well known to mark epithelial stem cells in continuously growing mouse incisors. Sox2 marks epithelial competence for tooth generation in mammals and reptiles.^10^, ^11^ Although the role of Sox2 in successional dental lamina of teeth is well identified, its relationship with intercellular junctional proteins has not been studied.

There are three main types of intercellular junctions: tight, adherens, and gap junctions. Tight junctions play important roles in regulating the development and normal functioning of cells.^12^, ^13^ As tight junctions enable proteins to recruit signalling proteins, tight junctions are involved in the regulation of cell proliferation, differentiation and many other cellular functions. Furthermore, tight junctions in developing tooth germs may play critical roles in morphogenesis and cell differentiation. In particular, tight junctions have also been observed in odontoblasts at later stages of tooth development.^14^ Claudins are the most important components of tight junctions. There are 24 claudin members in mammals.^15^ Although the expression of claudins has been identified,^16^ the functions of Claudins and their relationships with signalling molecules in tooth development have not been identified to date.

The basement membrane is a thin, sheet‐like extracellular matrix that separates the epithelium and mesenchyme and surrounds many cell types, including endothelial cells. The basement membrane plays a role in organogenesis by supporting cells and providing signals for cell proliferation, migration and differentiation.^17^ Laminin is a heterotrimeric glycoprotein consisting of three genetically distinct alpha, beta and gamma chains.^18^ The laminins are an important and biologically active part of the basal lamina, influencing cell differentiation, migration and adhesion.^19^ Furthermore, Laminin α5 is required for the proliferation and polarity of basal epithelial cells to play an important role in determining the size and shape of the tooth germ. Therefore, this study aimed to investigate the coexpression of Sox2 and Claudin10 in developing mouse teeth and juvenile gecko teeth and to study the role of Sox2 as a regulator of the proliferation of the successional dental lamina in tooth development through regulation of Claudin10 and laminin5. Thus, this study provides important insight into how Sox2 interacts with tight junctions and basement membranes in the proliferation of the dental epithelium and is essential for tooth replacement.

## 2 MATERIALS AND METHODS

All experiments were performed according to the guidelines of the Yonsei University College of Dentistry, Intramural Animal Use and Care Committee (2012‐0105).

### 2.1 Animals

Adult ICR mice (purchased from Koatech Co, Pyeongtaek, Korea) were housed in a temperature‐controlled room (22°C) under artificial lighting (lights on from 05:00 to 17:00) and 55% relative humidity with access to food and water ad libitum. Embryos were obtained from time‐mated pregnant mice. E0 was designated as the day on which the presence of a vaginal plug was confirmed. Embryos from each developmental stage (E13.5, E15.5 and E16.5) were used in this study.

### 2.2 RT‐qPCR (Real time‐quantitative Polymerase Chain Reaction)

For quantification of the levels of RNA, the tooth germs were microdissected at each stage (initiation, bud, cap and bell stage), were separated the epithelium and the mesenchyme each by Dispase II (Roche, Mannheim, Germany), and RNA was extracted with TRIzol reagent. After DNase I treatment, the RNA was purified with an RNeasy Kit (Qiagen, Hilden, Germany). RT‐qPCR was performed with a Thermal Cycler Dice^™^ Real Time System and SYBR Premix EX Taq^™^ (Takara, Kyoto, Japan) according to the manufacturer's instructions.

The primers used for amplification were as follows:

Sox2
Forward 5′‐CTGGACTGCGAACTGGAGAAG‐3′
Reverse 5′‐TTTGCACCCCTCCCAATTC‐3′
Claudin10
Forward 5′‐CAAAGTCGGAGGCTCAGATCA‐3′
Reverse 5′‐CAATCCCGGCCAAGCA‐3′
LamC2
Forward 5′‐GCCAAATTCCTCGGTAACCA‐3′
Reverse 5′‐CCACGCGGTAGTCAAAAGACA‐3′
Pcna
Forward 5′‐TGCTGACATGGGACACTTAAACTA‐3′
Reverse 5′‐CAATGCGAACATGCTTCCTCAT‐3′

### 2.3 Sox2‐expressing lentiviral vector treatment

Tooth germs were isolated from E15 mouse mandibles and then cultured in DMEM (Gibco, NH, USA) including 10% FBS and 1% penicillin and streptomycin at 37°C and 5% CO_2_ for 2 days. Lentivirus were produced following one hundred milliliters of concentrated Sox2‐expressing lentivirus was added to 1 mL of culture medium containing TransDux (SystemBiosciences, CA, USA).

### 2.4 Luciferase assay

PCR was used to amplify 1 kb of the Claudin10 promoter region including the Sox2‐binding sites (CACAATG) from −963 bp to +36 bp (primers used: sense 5′‐TGCGGTACCTCTGACCTCCACATGTAGT‐3′ and antisense 5′‐GCCAAGCTTGAAGGTGTTGGTACTGCAGA‐3′). These sequences were inserted into the pGL3‐Basic vector to construct a Claudin10‐luciferase reporter (pGL3‐Claudin10). After 24 hours, the Claudin10 promoter‐containing reporter plasmid and the pCDH‐Sox2‐T2A‐EGFP plasmid were cotransfected into human embryonic kidney 293T cells using FuGENE HD transfection reagent (Roche). An empty pGL3‐Basic plasmid; pRL‐TK, the Renilla luciferase vector and pCDH‐Sox2‐T2A‐EGFP were also cotransfected into cells to standardize the transfection efficiency. Luciferase assays were performed 48 hours post‐transfection using a dual‐luciferase assay system (Promega, WI, USA).

### 2.5 ChIP assay

Chromatin immunoprecipitation (ChIP) was performed using a Chromatin Immunoprecipitation Kit (Millipore) according to the manufacturer's instructions. Briefly, 293T cells transfected with pCDH‐Sox2‐GFP were treated with 1% formaldehyde to crosslink the proteins and DNA. The cell lysates were sonicated to shear chromosome to sizes of 200 to 500 bp. Equal aliquots of chromatin supernatants, into which 1 μg of either anti‐SOX2 (Abcam, CAM, UK) or anti‐IgG (the negative control) was added, were incubated overnight at 4°C with rocking. After reverse crosslinking of the protein/DNA complexes to free the DNA, PCR was performed using specific primers to amplify a 96 bp region (site 1) of the Claudin10 promoter region (primers: forward 5′‐CTGGTAGTCGCATGGTTCGT‐3′ and reverse 5′‐AGGGTTTTGATTTCGCAGAC‐3′).

### 2.6 IdU/BrdU and BrdU

Both IdU and BrdU were injected (both 70 μg/g body weight) into pregnant ICR mice at E13.5, E15.5 and E16.5 (n = 5) and into juvenile geckos (n = 5). The mandibles of the embryos were embedded in wax and sectioned at 4 μm thickness. We used mouse monoclonal anti‐BrdU (Becton Dickson Ltd., NJ, USA), which can recognize both IdU and BrdU, and rat monoclonal anti‐BrdU (Bio‐Rad, CA, USA). For the secondary antibodies, Alexa Fluor 488‐conjugated goat anti‐mouse (Invitrogen; dilution 1:200) and Alexa Fluor 555‐conjugated goat anti‐rat (dilution 1:200) antibodies were used. The cell cycles were determined according to a previously described formula.^20^

### 2.7 Immunofluorescence

The specimens were embedded in wax using conventional methods. Sections (4 μm thickness) of the specimens were boiled in 10 mmol/L citrate buffer (pH 6.0) for 20 minutes and cooled at RT for 20 minutes. The specimens were incubated with primary antibodies against Sox2 (R&D system, MN, USA; dilution 1:40), Claudin10 (Abcam, CAM, UK; dilution 1:100), Laminin5 (Abcam; dilution 1:100) and PCNA (Abcam; dilution 1:400) at 4°C overnight. The specimens were incubated with goat anti‐rabbit Alexa Fluor 488 (Abcam; dilution 1:200), donkey anti‐mouse Alexa Fluor 555 (Abcam; dilution 1:200) and donkey anti‐goat Alexa Fluor 647 (Abcam; dilution 1:200) antibodies. The sections were counterstained with DAPI (Molecular Probes, OR, USA; dilution 1:1000) and examined using a confocal laser microscope (LSM 510 META Ver. 3.2; Carl Zeiss, Oberkochen, Germany).

## 3 RESULTS

### 3.1 The expression patterns of Sox2, Claudin10 and Laminin5 during mouse tooth development

Ohazama A and Sharpe P examined expression patterns of Claudins in tooth development, especially those of Claudin10, which was expressed in the lingual epithelium of the developing tooth germ.^16^ Furthermore, *Sox2* is strongly expressed at the lingual side of the molars.^11^ However, although each of Sox2 and Claudin10 localization has been studied, co‐localization has not been studied yet.

**Figure 1:**
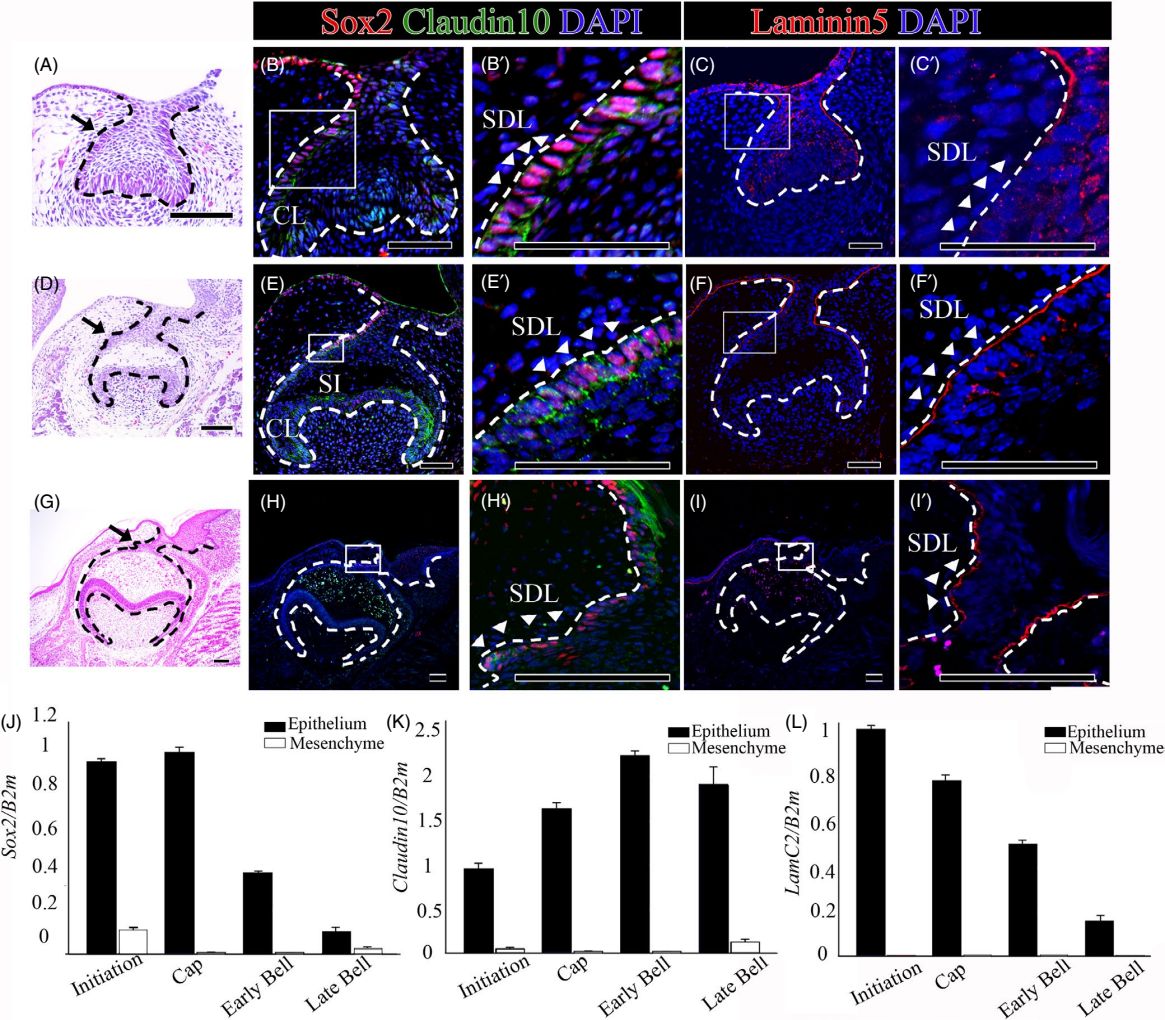
The expression patterns of Sox2, Claudin10 and Laminin5 during tooth development. (A, B, B’, C, C’) Cap stage tooth germs (E13.5), (D, E, E’, F, F’) early bell stage tooth germs (E15.5) and (G, H, H’, I, I’) late bell stage tooth germs (E18.5) were compared. (A, D, G) H&E staining, (B, B’, E, E’, H, H’) Sox2 and Claudin10 coexpression patterns and (C, C’, F, F’, I, I’) Laminin5 expression patterns in the frontal sections of tooth germs. (J, K, L) RT‐qPCR analysis of separated the oral epithelium and dental mesenchyme at the initiation, cap, early bell and late bell stages. (J) *Sox2* expression levels and (K) *Claudin10* expression levels in the oral epithelium and dental mesenchyme. (L) Laminin5 (*LamC2*) expression levels in the oral epithelium and dental mesenchyme. Scale bar = 100 µm, *SDL* successional dental lamina*, CL* cervical loop*, SI* stratum intermedium

To investigate the coexpression between Sox2 and Claudin10, immunofluorescence was performed from the cap stage to the late bell stage. At the cap and early bell stages, Sox2 and Claudin10 were colocalized on the lingual sides of the tooth germs (Figure 1A, B, B’, D, E, E’). Claudin10 was also expressed in other regions, such as the cervical loop and the stratum intermedium region. At the late bell stage, only Sox2 was expressed in the successional dental lamina, and Claudin10 expression was almost absent (Figure 1G, H, H’). RT‐qPCR revealed that the *Sox2* expression level was higher in the epithelium than in the mesenchyme. Furthermore, *Sox2* expression gradually decreased after the early bell stage (Figure 1J). *Claudin10* was expressed mainly in the epithelium, not in the mesenchyme, and was expressed at the highest level in the early bell stage (Figure 1K). The pattern of *Claudin 10* expression was not the same as that of *Sox2* expression. The reason for this difference was that Claudin 10 was expressed in other areas besides the successional dental lamina, which overlapped with areas of Sox2 expression in the developing tooth. Therefore, Sox2 and Claudin10 expression was colocalized on the lingual side of the dental epithelium, especially in the successional dental lamina region.

Laminin5 expression was only observed in the dental epithelium from the cap stage to the late bell stage. At the cap stage, Laminin5 was expressed in the dental epithelium, including in the successional dental lamina (Figure 1C,C’). Laminin5 expression was high both on the basal and the apical side of the basement membrane in the oral and dental epithelium at the early bell stage (Figure 1F,F’) and the late bell stage (Figure 1I,I’). RT‐qPCR revealed that the Laminin5 (*LamC2*) expression level was higher in the epithelium than in the mesenchyme. Furthermore, *LamC2* expression gradually decreased after the cap stage (Figure 1L).

### 3.2 The expression patterns of Sox2, Claudin10 and Laminin5 during continuous tooth replacement in juvenile geckos

**Figure 2:**
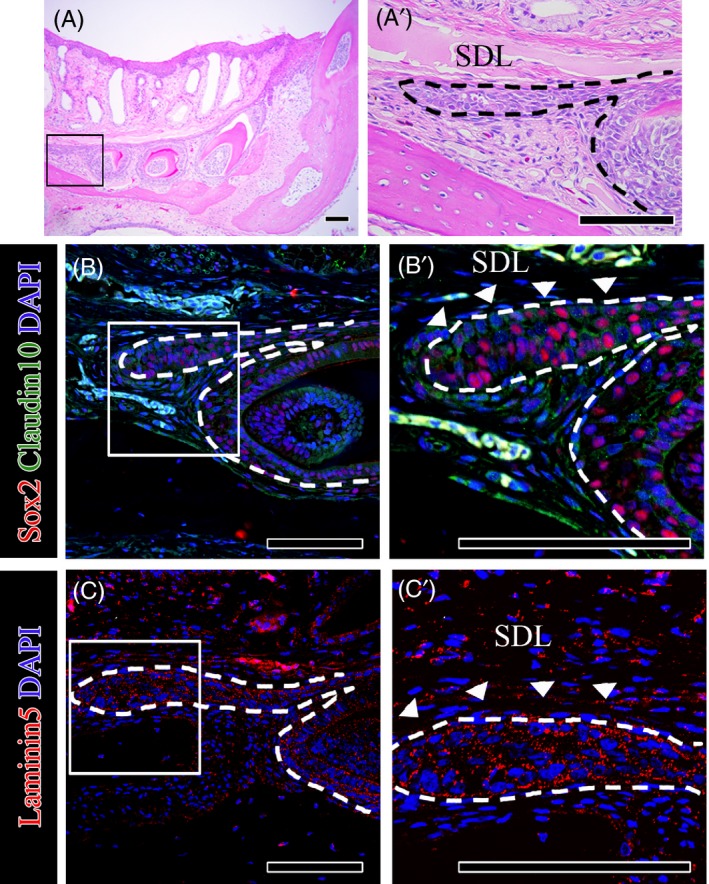
The expression patterns of Sox2, Claudin10 and Laminin5 during tooth development in geckos. (A, A’) H&E staining, (B, B’) Sox2 and Claudin10 coexpression patterns (C, C’) and Laminin5 expression patterns in the frontal sections of juvenile gecko tooth germs. Scale bar = 100 µm, *SDL* successional dental lamina

Continuous tooth replacement in geckos has been characterized, and putative dental stem cells are localized on the lingual side of the dental lamina.^21^, ^22^ We used the leopard gecko as a model of continuous tooth replacement to study Sox2 and Claudin10 (Figure 2A,A”). Sox2 and Claudin10 were colocalized in the successional dental lamina extending from the pre‐generation teeth (Figure 2B,B’). The results regarding the regional differences in Sox2 expression that appeared in the dental lamina are consistent with those of Jurri *et al.* 11 In this study, Sox2 expression was observed at the free end of the successional lamina, which actively proliferated to produce the next generation of teeth in juvenile geckos. Laminin5 was strongly expressed in the successional dental lamina of the juvenile gecko tooth germ (Figure 2C,C’).

### 3.3 Comparison of the cell cycle in the successional dental lamina between mice and geckos

**Figure 3:**
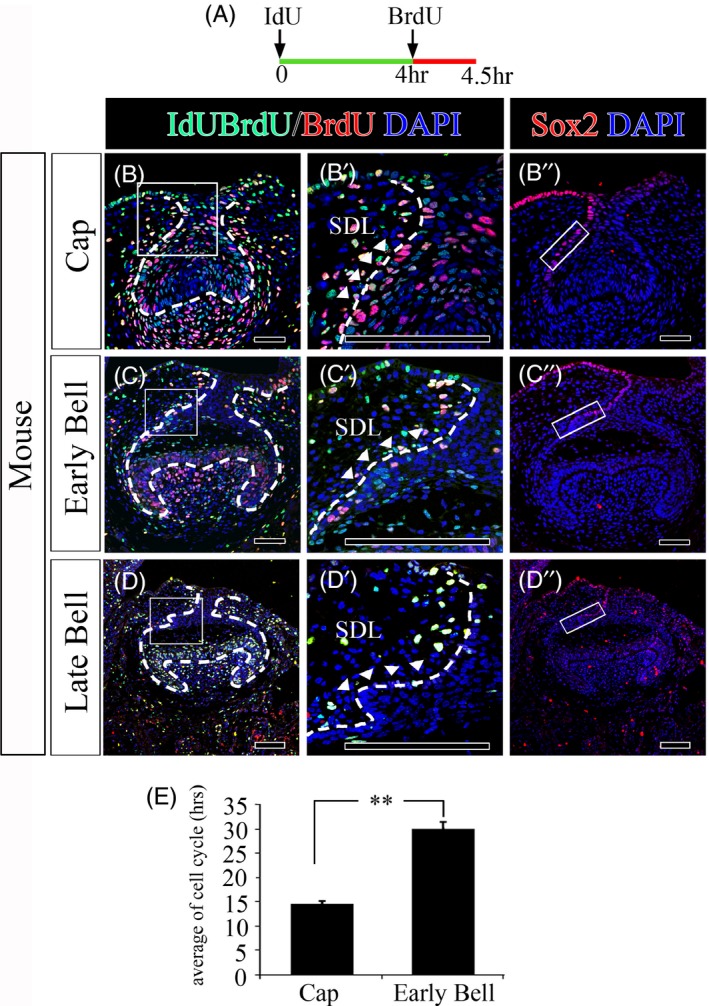
Cell cycle analysis of the successional dental lamina during mouse tooth development. A, The mouse IdU/BrdU injection schedule. IdU and BrdU staining of the successional dental lamina in the (B, B’) cap stage, (C, C’) early bell stage and (D, D’) late bell stage of the frontal sections of mouse tooth germs. Sox2 staining of the successional dental lamina in the (B’’) cap stage, (C’’) early bell stage and (D’’) and late bell stage of mouse tooth germs. E, Comparison of cell cycle in the successional dental lamina between the cap and early bell of mouse tooth germs. Scale bar = 100 µm, *SDL* successional dental lamina. The squares in (B’’, C’’, D’’) represent the areas where the cell cycles were calculated. The epithelial region that is considered to be a successional dental lamina among the sites where Sox2 is expressed. Scale bar = 100 µm, *SDL* successional dental lamina

To understand the cellular mechanism in the successional dental lamina, we analysed and compared the cell cycles during developing mouse teeth. In this study, the cell cycle of the successional dental lamina in developing mouse teeth was examined by injection of both IdU and BrdU at the early cap stage, the early bell stage and the late bell stage. Based on the cell cycle in the inner dental epithelium,^20^ BrdU was injected 4 hours after IdU was injected (Figure 3A). The mice were sacrificed 30 minutes after BrdU injection. The cell cycle of the successional dental lamina was calculated based on the site considered to be successional dental lamina where Sox2 is expressed only in epithelium. The cell cycle of the successional dental lamina where Sox2 is expressed in the cap stage (Figure 3B,B’ and B”) and the early bell stage (Figure 3C,C’ and C”) were calculated to be 14 hours 25 minutes and 30 hours, respectively, on average, after several trials. With regard to the cell cycle of the successional dental lamina at the late bell stage, the expression of IdU and BrdU did not appear, and the cell cycle was not calculated (Figure 3D,D’ and D”). In the successional dental lamina of the mouse tooth, the cell cycle gradually slowed down and eventually did not proliferate after the late bell stage (Figure 3E). Therefore, reduced expression of Sox2 might regulate the cell cycle in the successional dental lamina during mouse tooth development.

### 3.4 Comparison of cell proliferation in the successional dental lamina between mice and geckos

**Figure 4:**
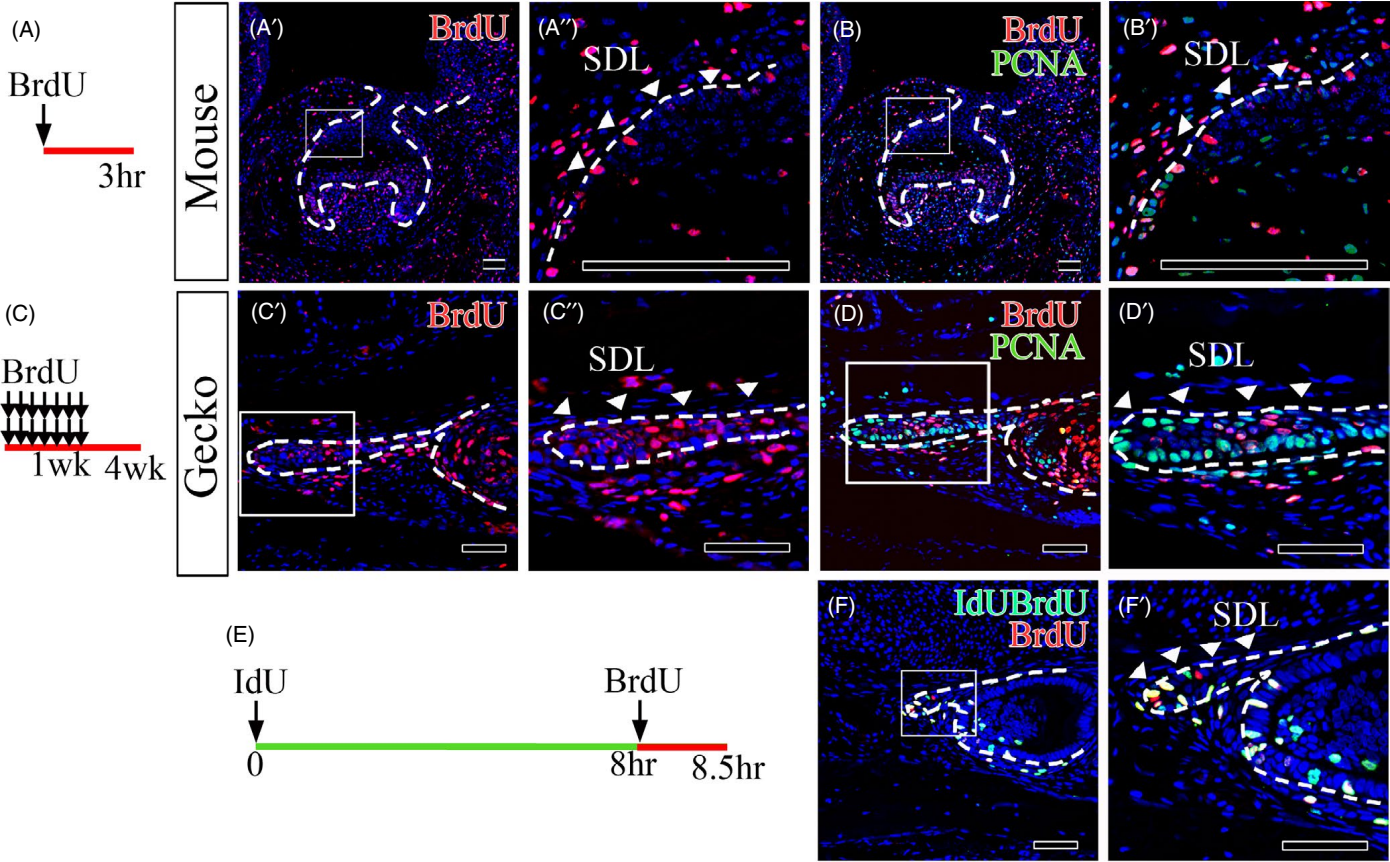
Cell proliferation in the successional dental lamina between the frontal sections of mouse and gecko tooth germs. A, The mouse BrdU injection schedule and the (C) Gecko BrdU injection schedule. BrdU staining in (A’, A’’) 3 h BrdU‐injected mouse tooth germs at early bell stage, (C’, C’’) tooth germs from 1‐week BrdU‐injected geckos sacrificed 4 weeks after the first injection. BrdU and PCNA staining in (B, B’) early bell stage mouse tooth germs and (D, D’) gecko tooth germs. E, The gecko IdU/BrdU injection schedule. IdU and BrdU staining of the successional dental lamina in the (F, F’) successional dental lamina in juvenile gecko tooth. Scale bar = 100 µm, *SDL* successional dental lamina

To further determine whether the dental epithelium of mice and geckos contains slow‐cycling cells, we performed a BrdU pulse‐chase experiment on mice and geckos. Handrigan et al performed experiments and showed that label‐retaining cells (LRCs) were identified in juvenile geckos.^21^ In this study, we compared BrdU pulse chases between mouse teeth at the early bell stage and the teeth of juvenile geckos. When we pulsed mice with BrdU for 3 hours, BrdU was incorporated into only one cell or two cells on average (Figure 4A,A’ and A”). Proliferation is low in the successional dental lamina during mouse tooth development. Next, we analysed BrdU retention in geckos chased for 4 weeks to label proliferating cells. Juvenile geckos injected twice per day with BrdU for 1 week to label proliferating cells were sacrificed after the 4‐week chase period. After a 4‐week chase, the label‐retaining cells were randomly distributed in the successional dental lamina (Figure 4C,C’ and C”).

Furthermore, in this study, as BrdU/PCNA‐double positive (BrdU + PCNA+) cells represent putative slow cycling stem/progenitor cells,^23^ we identified BrdU + PCNA+cells in the dental epithelium of mice and geckos. Very few BrdU + PCNA+cells were found in successional dental lamina of mice (Figure 4B,B’). On the other hand, in the successional dental lamina of juvenile gecko, many BrdU + PCNA+stem/progenitor cells existed in the middle of the successional dental lamina. At the end of the successional dental lamina, we found many proliferating transit amplifying cells, such as BrdU‐PCNA + cells (Figure 4D,D’). Cell cycle with IdU and BrdU injection was calculated at succession dental lamina of juvenile gecko. We tried to perform injections with the same time intervals as those used in mice; however, we could not find sufficient IdU/BrdU‐labelled cells (L_cells_) (data not shown). Therefore, we decided to use a time interval between IdU and BrdU injection of 8 hours. The injected geckos were sacrificed 30 minutes after BrdU injection (Figure 4E). The average cell cycle was 44 hours and 11 minutes (Figure 4F,F’). We conclude that the juvenile gecko successional dental lamina contains slow‐cycling with a cell cycle of about 45 hours.

### 3.5 Sox2 overexpression enhances cell proliferation in dental epithelium

**Figure 5:**
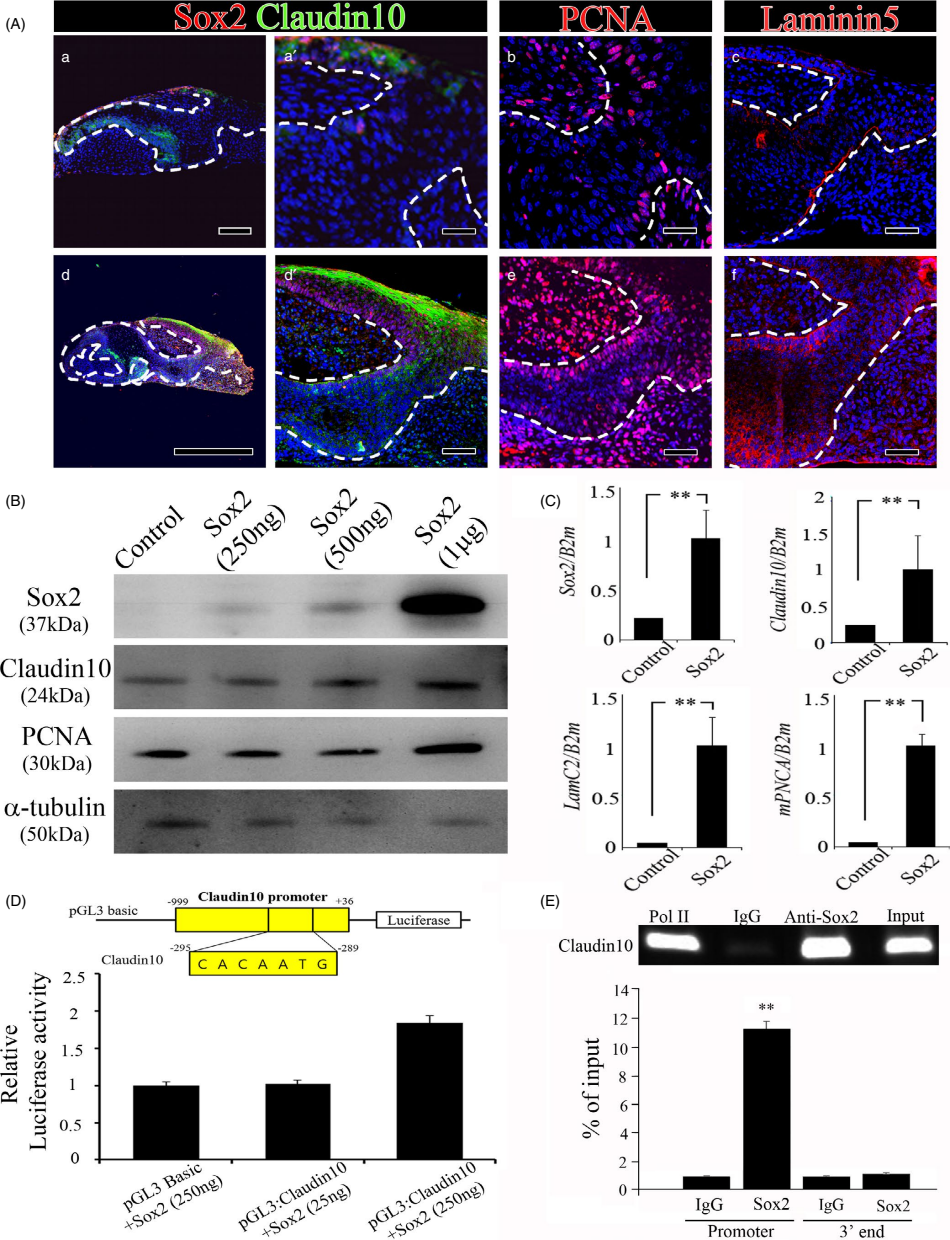
Sox2 overexpression at the early bell stage and in 293T cells. Mouse tooth germs at early bell stage were cultured for 2 days with a (A‐a, a’, b, c) control or (A‐d, d’, e, f) Sox2‐overexpressing lentivirus. Sox and Claudin10 coexpression patterns in the (A‐a, a’) control and (A‐d, d’) Sox2 overexpression groups, PCNA expression patterns in the (A‐b) control and (A‐e) Sox2 overexpression groups, and Laminin5 expression patterns in the (A‐c) control and (A‐f) Sox2 overexpression groups were analysed. B, Western blot and (C) RT‐qPCR analyses of control and Sox2 overexpression in 293T cells. D, Relative luciferase activity and (E) ChIP assays were used to confirm the binding of Sox2 with the Claudin10 promoter. Scale bar = 100 µm (A‐a’, b, c, d’, e, f), 200 µm (A‐a) or 1 mm (A‐d)

To investigate whether Sox2 regulates cell proliferation and interacts with Claudin10 and Laminin5, Sox2 was overexpressed in the early bell stage (in which Sox2 is normally expressed at low levels) using a lentiviral system, and the samples were cultured for 2 days. The lentivirus Sox2 overexpression resulted in a very thicker tooth dental epithelium and a very longer dental stalk (Figure 5A). After 2 days in vitro culture with Sox2 overexpression lentivirus, Sox2 and Claudin10 were overexpressed in the dental lamina and dental stalk (Figure 5A‐A,A’,d,d’). To determine the effect on proliferation in the dental epithelium, PCNA staining was performed in both the Sox2 overexpression group and the control group. Higher rates of PCNA‐positive cells were observed in dental stalks with epithelial hyperplasia (Figure 5A‐B,E). Laminin5 was more strongly expressed in dental stalks in the Sox2 overexpression group group than in the control group (Figure 5A‐C,F).

Following Sox2 overexpression in 293T cells for 2 days, Western blotting and RT‐qPCR were used to confirm the transfection efficiency of the lentiviral system using an antibody against Sox2. Sox2 expression was observed to increase in a dose‐dependent manner (Figure 5B,C). Furthermore, the translational levels and transcriptional levels of Claudin10 and PCNA were increased in the Sox2 overexpression group compared with the control group (Figure 5B,C). Thus, Sox2 enhances the proliferation of the dental epithelium by regulating Claudin10. To determine whether Sox2 upregulates Claudin10 expression directly, 293T cells were cotransfected with various doses of pGL3‐Basic:Sox2 expression vectors and with Claudin10 containing the Sox2‐binding site. The relative luciferase activity was significantly upregulated in the cells transfected with the Sox2 expression constructs (250 ng) compared with those transfected with the pGL3‐Basic constructs (Figure 5D). The Claudin10 promoter possesses the typical binding motifs for Sox2. As a control, the 3' end of the Claudin10 gene was used. ChIP assays with 293T cells overexpressing Sox2 confirmed that Sox2 interacted with Claudin10 promoter (Figure 5E). Therefore, these findings provide direct evidence showing that Sox2 regulates the direct induction of Claudin10 transcription for dental epithelium proliferation.

## 4 DISCUSSION

The teeth of different species have different regenerative capacities. Reptiles replace their teeth continuously throughout their lives, whereas in mammals, tooth replacement is restricted to one round.^11^ During mammalian evolution, replacement capacity has been reduced, whereas the complexity of tooth shapes has increased. The capacity for tooth replacement is believed to reside in the dental lamina and successional dental lamina. Label‐retaining putative stem cells have been localized in the successional dental lamina in species with lifelong tooth replacement, including the leopard gecko (*Eublepharis macularius*),^21^ the alligator 24 and the zebrafish (*Danio rerio*).^25^

Sox2‐positive stem cells give rise to all epithelial cell lineages of the incisor, are associated with tooth renewal in general, and have been proposed to include the stem cells for all dental epithelial tissues.^10^, ^26^ Tight junctions regulate the passage of molecules through the paracellular pathway in epithelial cells.^27^ Claudins are considered core components of tight junction and determine the epithelial permeability of small molecules.^28^ Among the Claudin family, Claudin10 has been identified to exhibit localized expression in the lingual basal epithelium of the developing tooth germ.^16^ In this study, Sox2 and Claudin10 were colocalized on the lingual side of the tooth germ during mouse embryonic tooth development; however, the expression level in the successional dental lamina was decreased after the bell stage. On the other hand, the coexpression of Sox2 and Claudin10 was sustained in the successional dental lamina of the juvenile gecko tooth. This results suggest that Sox2 and Claudin10 regulate to maintain the successional dental lamina during tooth development. Furthermore, with a reporter assay and a ChIP assay, Claudin10 promoter activity was found to be increased by the Sox2 expression construct, providing evidence that Sox2 directly regulates Claudin10 transcription to regulate to maintain the successional dental lamina and proliferation of dental epithelium during tooth development in gecko.

Epithelial region in the pleurodont and acrodont of Beard dragon was suggested that BrdU+PCNA+ cells were present among label‐retaining putative stem cells.^23^ In the present study, BrdU+PCNA+ cells were observed in the successional dental lamina of the juvenile gecko tooth. Additionally, at the free end of the successional dental lamina, there were many PCNA‐positive and Sox2‐positive cells. In this study, the cell cycle calculated in the successional dental lamina where Sox2 was expressed after IdU and BrdU injection was decreased and eventually was not able to be calculated after the late bell stage in mouse tooth germs, and proliferating cells could no longer be detected in the successional dental lamina. On the other hand, many slow‐cycling stem/progenitor cells (BrdU+PCNA+) in the middle of successional dental lamina and proliferating transient amplifying cells in the free end of successional dental lamina were shown in juvenile gecko teeth. Furthermore, recent studies suggested a relationship between Claudins and cell proliferation. Overexpression of Claudin2 promotes self‐renewal within colorectal cancer stem‐like cells.^29^ Claudin18 suppresses the abnormal proliferation and motility of lung epithelial cells.^30^ We showed Sox2 and Claudin10 coexpression and BrdU label retention studies on juvenile geckos. Therefore, we provide strong evidence that Sox2 and Claudin 10 regulate not only population of slow cell cycling cells but also population of proliferating cells in the lingual portion of the gecko dental lamina.

It has been suggested that Sox2 overexpression in aggressive human breast carcinomas promotes β‐catenin‐stimulated proliferation.^31^, ^32^ Furthermore, inactivation of Sox2 using Ptx2‐Cre leads to dental defects due to impaired stem cell proliferation and defective dental epithelial cell differentiation.^33^ Furthermore, siRNA knock‐down of Sox2 results in arrested tooth morphogenesis in the second molar, reduced cell motility and increased apoptosis during tooth development.^34^ Knock‐down of Sox2 significantly inhibits the multipotentiality of mesenchymal stem cells and cell proliferation.^35^ Sox2 regulates the differentiation of endodermal progenitor cells of the tongue into taste bud sensory cells vs keratinocytes.^36^ In our present study, overexpression of Sox2 at the early bell stage in mouse tooth germs promoted proliferation in the dental epithelium and hyperplasia of dental stalks and regulated the progenitor states of dental epithelial cells.

Abnormal expression of laminin5 contributes to the aberrant proliferation of cyst epithelial cells in polycystic kidney disease.^37^ A cell signalling pathway involving laminin5 can regulate epithelial cell proliferation.^38^ Furthermore, in mice, disrupted laminin staining at the basement membrane on the aboral side of the tooth leads to the process of dental lamina loss.^2^ We showed that *LamC2* expression was decreased after the cap stage in the developing tooth germ in mice; however, Laminin5 expression was maintained in the successional dental lamina in the teeth of juvenile geckos. Additionally, upon Sox2 overexpression in the tooth germ at the late bell stage and in HEK 293T cells, Laminin5 expression was highly increased. Therefore, in mice, a decrease in Sox2 expression regulates Claudin10 and Laminin5 and leads to stop cell proliferation in the successional dental lamina of the tooth germ during mouse embryonic tooth development. In geckos, retained expression of Sox2 in the successional dental lamina leads to maintenance of the expression of Claudin10 and Laminin5 and to continuous proliferation in the successional dental lamina for tooth replacement.

Altogether, our study reveals coexpression patterns, cell proliferation patterns and the relationship among Sox2, Claudin10 and Laminin5 in the successional dental lamina in mice compared with geckos. This study reveals the regulatory mechanisms of the cell cycle and cell proliferation in the dental epithelium. Based on our findings, we conclude that Sox2, Claudin10 and Laminin5 play significant roles in dental epithelial proliferation along the dental lamina.

## CONFLICT OF INTEREST

The authors have no conflicts of interest to declare.

## AUTHOR CONTRIBUTION

HSJ and EJK designed the study: HSJ, EJK, SYJ, ZW and SZhang involved in data collection & analysis.

## Data Availability

Research data are not shared.
